# Evaluation of the inhibition of carbohydrate hydrolysing enzymes, antioxidant activity and polyphenolic content of extracts of ten African *Ficus* species (Moraceae) used traditionally to treat diabetes

**DOI:** 10.1186/1472-6882-13-94

**Published:** 2013-05-04

**Authors:** Oyinlola O Olaokun, Lyndy J McGaw, Jacobus N Eloff, Vinny Naidoo

**Affiliations:** 1 Phytomedicine Programme, Department of Paraclinical Sciences, University of Pretoria, Private Bag X04, Onderstepoort 0110, South Africa; 2 Biomedical Research Centre, Faculty of Veterinary Sciences, University of Pretoria, Onderstepoort 0110, South Africa; 3 Permanent address: Federal Institute of Industrial Research Oshodi (FIIRO), Ikeja, Nigeria

**Keywords:** African *Ficus* species, *Ficus lutea*, Diabetes, α-amylase inhibitory activity, α- glucosidase inhibitory activity, Antioxidant activity, Polyphenol content

## Abstract

**Background:**

Some *Ficus* species have been used in traditional African medicine in the treatment of diabetes. The antidiabetic potential of certain species has been confirmed *in vivo* but the mechanism of activity remains uncertain. The aim of this study was to determine the activity and to investigate the mechanism of antidiabetic activity of ten selected *Ficus* species through inhibition of α-amylase and α-glucosidase activity, and the possible relationship between these activities, the total polyphenolic content and the antioxidant activity.

**Methods:**

Dried acetone leaf extracts were reconstituted with appropriate solvents and used to determine total polyphenolic content antioxidant activity, α-amylase and α-glucosidase inhibitory activity.

**Results:**

The crude acetone extract of *F. lutea* had the highest polyphenolic content (56.85 ± 1.82 mg GAE/g of dry material) and the strongest antioxidant activity with a TEAC value of 4.80 ± 0.90. The antioxidant activity of the acetone extracts of the *Ficus* species may not be ascribed to total polyphenolic content alone. The crude extract at a concentration of 0.5 mg/ml of *F. lutea* (64.3 ± 3.6%) had the best α-glucosidase (sucrase) inhibitory activity. The EC50 of *F. lutea* (290 ± 111 μg/ml) was not significantly different from that of *F*. *sycomorus* (217 ± 69 μg/ml). The α-amylase inhibitory activity of *F. lutea* (95.4 ± 1.2%) at a concentration of 1 mg/ml was the highest among the *Ficus* species screened. The EC50 for *F. lutea* (9.42 ± 2.01 μ g/ml), though the highest, was not significantly different (*p* < 0.05) from that of *F. craterostoma* and *F. natalensis*. It was apparent that the crude acetone extract of *F. lutea* is a partially non-competitive inhibitor of α-amylase and α-glucosidase. Based on correlation coefficients polyphenolics may be responsible for α-glucosidase activity but probably not for α-amylase activity.

**Conclusion:**

Antidiabetic activity potential via inhibition of α-amylase and α-glucosidase was discovered in *Ficus lutea* which has not been previously reported. The acetone extract of the leaves was high in total polyphenolic content and antioxidant activity, and was a potent inhibitor of α-amylase activity. Research is underway to isolate the active compound(s) responsible for the antidiabetic activity and to confirm the *in vitro* antidiabetic activity and to investigate *in vitro* toxicity.

## Background

Diabetes mellitus is an endocrine disease characterised by chronic hyperglycaemia with the disturbance of carbohydrate, fat and protein metabolism resulting from defects in insulin secretion, insulin action or both [1], and is typically associated with the failure of pancreatic β-cells. There are two major types of diabetes mellitus - type 1 and type 2. In type 1 diabetes, or insulin dependent diabetes mellitus, the body has little or no insulin secretory capacity and depends on exogenous insulin to prevent metabolic disorders and death. In type 2 diabetes, a non-insulin dependent diabetes mellitus, the body retains some endogenous insulin secretory capability; however, their insulin levels are low relative to their blood glucose levels and/or there is a measure of insulin resistance. Type 2 diabetes is the most prevalent form of the disease, accounting for 90-95% of cases [2,3].

Obesity, a sedentary lifestyle and a diet high in carbohydrate and fats are known risk factors for type 2 diabetes [4]. Globally, 171 million people were diagnosed with the disease in 2000, and it has been predicted that the prevalence will grow to 366 million by 2030 if no intervention action is taken [5]. In sub-Saharan Africa the disease is also considered to be an important emerging disease partly due to changes in diet and lifestyle of the people (a highly refined diet coupled to reduced physical activity). This is brought about by westernization, urbanization and mechanization, i.e. the disease which was once associated with only the affluent has increased in prevalence from 1% to 20% while in South Africa it is 4% to 6% [3,6,7].

The prolonged hyperglycaemia that results in diabetic patients has been speculated to induce oxidative stress through the excessive generation of free radicals which may impair endogenous antioxidant defence [8]. The stabilization of blood glucose in diabetic patients is therefore important to prevent these hyperglycaemic complications [9]. The main therapeutic approach used to achieve this objective in the diabetic patient is to stimulate insulin release, increase the number of glucose transporters, inhibit gluconeogenesis or reduce the absorption of glucose [10]. Another therapeutic approach is to decrease the post-prandial hyperglycaemia [11], which is achieved with the enzyme inhibitors such as acarbose, voglibose and miglitol [12], which function by retarding the action of the gastrointestinal carbohydrate hydrolysing enzymes α-amylase and α-glucosidase. As a result, these substances delay carbohydrate digestion thereby decreasing the rate of glucose absorption, i.e. they blunt the post-prandial plasma glucose rise [13]. While effective, the gastrointestinal side effects of acarbose, which includes bloating, abdominal discomfort, diarrhoea, and flatulence make them less attractive as therapeutic agents. New anti-diabetic compounds which function by this mechanism but devoid of side effect are therefore desirable [12,14].

*Ficus* is a genus of about 800 species of woody trees, shrubs and vines in the family Moraceae. They are found in all tropical habitat types, with about 100 species occurring in Africa and the surrounding islands [15]. Several species of the genus *Ficus* are used traditionally in a wide variety of ethnomedical remedies all over the world [16,17]. They have long been used in folk medicine as antidiabetic, anthelmintic, hypotensive, mild laxative, antirheumatic, digestive and anti-dysentery drugs [18,19]. From previous studies, these plants are known to have chemical constituents such as triterpenes, sterols, polyphenols, flavonoids, coumarins, alkaloids and other metabolites [20]. Polyphenolics are among the naturally occurring antidiabetic agents [21], which may function via various biological effects, of which one is the inhibition of hydrolysing enzymes [22]. Polyphenolic compounds are also one of the major constituents of medicinal compounds which act as free radical scavengers and antioxidants. Free radicals can react with biological molecules, leading to cell and tissue injuries and pathological events. Therefore the discovery of polyphenolic compounds with a potential to inhibit the activity of digestive enzymes and having excellent antioxidant activity with low adverse effects is important for the treatment of diabetes.

Twelve *Ficus* species have antidiabetic activity with glucose lowering activity in alloxan or streptozotocin induced diabetic laboratory animals. These are *Ficus benghalensis* L. [23], *Ficus carica* L. [24], *Ficus racemosa* L. [25], *Ficus hispida* L.f. [26], *Ficus microcarpa* L.f. [27], *Ficus religiosa* L. [28], *Ficus thonningii* Blume [29], *Ficus glumosa* Delile. [30]*Ficus arnottiana* (Miq.)Miq. [31], *Ficus glomerata* Roxb. [32], *F. sycomorus* L. [33] and *F. deltoidea* Jack [34]. Several *Ficus* species are also used traditionally to treat diabetes and other ailments (Table 1). Four of the *Ficus* species used in the current study have *in vivo* blood glucose lowering potential (Table 2). With the mechanism(s) of action being unknown, we investigate the α-amylase and α-glucosidase enzymes inhibitory activity of the acetone extracts of the leaves of the ten *Ficus* species (Table 1). Acetone was selected as extractant because it dissolves many hydrophilic and lipophilic components from plants [48], it is volatile and has low toxicity for use in bioassays [49]. Furthermore acetone does not extract sugars, which if present (as would be the case with water and alcoholic extracts) would complicate α-amylase and α-glucosidase inhibitory assays [50].

**Table 1:** Previous reports on the traditional uses of the selected *Ficus *species *F. religiosa is a naturalized exotic from China

**Species**	**Section**	**Sub-section**	**Parts used**	**Uses**	**Reference**
*F. capreifolia* Delile (sandpaper fig)	sycidium	-	Leaf, root	Infections, vulnerary schistosomiasis	[35]
*F. cordata* Thunb. (Namaqua rock fig)	urostigma	urostigma	Bark stem	Treatment of diarrhoea, tuberculosis	[36]
*F. craterostoma* Mildbr. & Burret. (forest fig)	galoglychia	chlamydodorae	leaves	Treatment of stomach ache	[37]
*F. glumosa* Delile (hair rock fig)	galoglychia	platyphyllae	Leaves, stem/bark	Treatment of skin diseases, diabetes,	[30,38]
*F. lutea* Vahl. (giant-leaves fig)	galoglychia	galoglychia	Leaves, bark	Treatment of sores, boils, madness, rabies	[39,40]
*F. natalensis* Hochst. (coastal strangler fig)	galoglychia	chlamydodorae	Leaf, fruits, bark	Treatment of skin infections, colic, venereal diseases	[18,38,41]
*F. polita* Vahl. (heart-leaved fig)	galoglychia	caulocarpae	Leaves, fruits, bark, root	Dyspepsia, infectious diseases, abdominal pain, diarrhoea	[42]
*F. religiosa* L. (bo tree or sacred fig)	*-	*-	Leaves, bark/stem	Treatment of ulcers, bacterial infections, asthma, diabetes	[43,44]
*F. sycomorus* L. (sycamore fig)	sycomorus	sycomorus	Stem/bark, leaves, root,	Treatment of tumours, inflammation, mental madness, pain, bacterial infections, diabetes	[45-47]
*F. thonningii* Blume (bark-cloth fig)	galoglychia	chlamydodorae	Leaves, fruits	Bronchitis, urinary tract infection, epilepsy, diabetes	[41,47]

**Table 2:** ***Ficus *species demonstrated to have blood glucose lowering potential *in vivo***

**Species**	**Part used**	**Solvent for extraction**	**Effective dose (mg/kg) *in vivo***	**References**
*Ficus glumosa*	stem/bark	methanol	65	[30]
*Ficus religiosa*	bark	water	50 and 100	[28]
*Ficus sycomorus*	Stem/bark	methanol	250	[33]
*Ficus thonningii*	stem/bark	ethanol	120 and 240	[29]

## Methods

### Materials and chemicals

Dimethyl sulphoxide (DMSO), acetone, methanol and Whatman No. 1 filter paper were purchased from Merck (South Africa). Folin-Ciocalteu reagent, gallic acid, sodium carbonate (Na_2_CO_3_), potassium persulphate, ABTS [2, 2–azinobis-(3- ethylbenzothiazoline-6-sulfonic acid)], potato starch, porcine pancreatic α- amylase enzyme (type VI), sodium chloride, sodium phosphate, 3, 5-dinitrosalicylic acid (DNS), sodium potassium tartrate, sodium hydroxide, acarbose, rat intestinal acetone powder, potassium phosphate, sucrose, TRIS (hydroxymethyl) aminomethane, hydrogen chloride (HCl), the glucose oxidase kit (GAGO 20) and Trolox (a synthetic water soluble vitamin E analogue) were sourced from Sigma (South Africa). The absorbance measurements were read using a microtitre plate reader (Versamax, Molecular Devices).

### Plant material

The leaves of ten *Ficus* species were collected at the Manie van der Schijff Botanical Garden (University of Pretoria), South Africa in February 2009, and voucher specimens were conserved in the HGWJ Schweikerdt Herbarium of the University of Pretoria. The names of plant species and voucher numbers are as follow: *Ficus capreifolia* Delile *-* PRU 33124; *Ficus cordata* Thunb *-* PRU 35501; *Ficus craterostoma* Warb. ex Mildbr. & Burret.- PRU 48293; *Ficus glumosa* Delile - PRU 38554, *Ficus lutea* Vahl.*-* PRU 074568; *Ficus natalensis* Hochst - PRU 078082; *Ficus polita* Vahl - PRU 35945; *Ficus religiosa* L. - PRU 115415; *Ficus sycomorus* L. - PRU 066173; and *Ficus thonningii* Blume - PRU 57036. The leaves were separated from the stems and dried at room temperature. The dried plant materials were milled to a fine powder in a Macsalab mill (Model 200 LAB, Eriez® Bramley) and stored at room temperature in closed glass bottles in the dark until extracted.

### Preparation of plant extracts

The powdered plant material (2 g) was extracted with acetone (20 ml) in polyester centrifuge tubes using a shaker (Labotec model 20.2) at room temperature for 30 min. The extracts were centrifuged and filtered through Whatman No. 1 filter paper. This procedure was repeated three times on the same plant material to ensure that all possible compounds were extracted. The filtered extracts of each species were transferred into pre-weighed glass vials and solvent was left to evaporate at room temperature under a stream of cold air. The percentage yield was expressed on the air-dried mass of the leaves. The dried crude acetone extracts obtained were dissolved in appropriate solvent and used for analyses of total polyphenolics, antioxidant activity and *in vitro* α-amylase and α-glucosidase inhibition.

### Total polyphenolic content

The amount of total polyphenolics was determined using the method described by Djeridane et al. [51]. This method depends on the reduction of Folin-Ciocalteu reagent (a 2 N phosphomolybdic-phosphotungstic reagent) by phenols to a mixture of blue oxides. The commercial reagent was diluted 10-fold with double-distilled water. A standard curve was developed using gallic acid. Different concentrations of gallic acid were prepared in 80% methanol in a 96-well microtitre plate, and their absorbance immediately recorded at 760 nm. The crude acetone extracts were dissolved in 80% methanol to a final concentration of 1 mg/ml. The plant extract (100 μl of the 1 mg/ml) was dissolved in 500 μl (1/10 dilution) of the Folin-Ciocalteu reagent. After the addition of 1000 μl of distilled water the mixture was allowed to stand for 1 min at room temperature, where after 1500 μl of a 20% Na_2_CO_3_ solution was added. The final mixture was shaken, followed by an incubation period of 1 h in the dark at room temperature. The absorbance of all samples was measured at 760 nm. All analyses were performed in triplicate and repeated three times. The results are expressed in mg of gallic acid equivalent (GAE) per gram of dry weight of plant material.

### Trolox equivalent anti-oxidant capacity (TEAC)

The total antioxidant activity was estimated using the Trolox equivalent capacity (TEAC) test [52]. In this assay, the relative capacity of antioxidant to scavenge the ABTS ^+^ [2, 2–azinobis- (3-ethylbenzothiazoline-6-sulfonic acid)] radical compared to the antioxidant potency of Trolox (6–hydroxyl–2,5,7,8-tetramethlchromane-2-carboxylic acid), a synthetic water soluble vitamin E analogue, was measured.

The ABTS ^+^ radical was produced by reacting 7 mM ABTS aqueous solution with 2.4 mM potassium persulphate in the dark for 12–16 h at room temperature. Prior to assay, the pre- formed ABTS ^+^ radical was diluted in methanol (about 1:89 v/v) and equilibrated to give an initial absorbance (Ai) of 0.700 (±0.02) at 734 nm. The crude acetone extracts were made up in methanol to a concentration of 0.0625 mg/ml to 1 mg/ml and the Trolox was made up to a concentration of 0.0625 mg/ml to 0.5 mg/ml in methanol. The crude acetone extract or Trolox standard (20 μl) was added to the ABTS ^+^ radical (180 μl) and the mixture allowed to react for 6 minutes, and the absorbance (A) read at 734 nm in a spectrophotometer at one- minute intervals. Appropriate solvent blanks were included in each assay. The percentage change in absorbance was calculated for each concentration of extracts and Trolox. To calculate the TEAC, the gradient of the plot of the percentage inhibition of absorbance versus concentration plot for the extract under investigation is divided by the gradient of the plot for Trolox [52]. As a result, the TEAC of a substance is a ratio value and has no unit. All assays were carried out in triplicate and repeated three times. The percentage change in absorbance (A) was measured as follows;

$$\begin{array}{l} \%\;\mathrm{change}\;\mathrm{in}\;A=\left(\frac{A_{i}{\;\mathrm{ABTS}}^{+}\left(734\mathrm{nm}\right)-{\mathrm{New}\;\mathrm{mean}\;A\;\mathrm{ABTS}}^{+}}{{\;A}_{i}{\;\mathrm{ABTS}}^{+}\left(734\mathrm{nm}\right)}\right)\\ \;\times 100 \end{array}$$

### α-Amylase inhibition assay

An adapted α-amylase inhibition assay as described by Ali et al. [50] was utilised. The dried crude acetone extracts were re-dissolved in DMSO to a concentration of 20 mg/ml and used for the α-amylase inhibition assay. Ice cold porcine pancreatic α-amylase solution (200 μl) at 4 unit/ml (type VI) was pre-incubated with 40 μl of crude acetone extracts and 160 μl of distilled water, and mixed in a screw-top plastic tube. The reaction was started by the addition of 400 μl of potato starch (0.5%, w/v) in 20 mM phosphate buffer (pH 6.9) containing 6.7 mM sodium chloride, and thereafter incubated at 25°C for 3 min. Final concentrations in the incubation mixture were plant extract (1 mg/ml), 0.25% (w/v) starch and 1 unit/ml enzyme. An aliquot of the mixture (200 μl) was removed and placed into a separate tube containing 100 μl DNS colour reagent solution (96 mM 3, 5-dinitrosalicylic acid, 5.31 M sodium potassium tartrate in 2 M NaOH) and placed into an 85°C water bath. After 15 min, this mixture was removed from the water bath, cooled and diluted with 900 μl distilled water. α-Amylase activity was determined by measuring the remaining starch content by measuring the absorbance of the mixture at 540 nm. Control incubations, representing 100% enzyme activity were conducted by replacing the plant extract with DMSO (40 μl). For the blanks (negative controls) the enzyme solution was replaced with distilled water and the same procedure was carried out as above. Acarbose was used as positive control (acarbose 1 mg/ml: α-amylase 1 unit/ml). The α-amylase inhibition activity was expressed as;

$$\begin{array}{l} \%\;\mathrm{Inhibition}=100\times \left(\frac{\Delta A_{\mathrm{Control}}-\Delta A_{\mathrm{Sample}}}{\Delta A_{\mathrm{Control}}}\right)\\ \;\Delta A_{\mathrm{Control}}=A_{\mathrm{Test}}-A_{\mathrm{Blank}}\\ \;\Delta A_{\mathrm{Sample}}=A_{\mathrm{Test}}-A_{\mathrm{Blank}} \end{array}$$

### α- Glucosidase inhibition assay

A commercial rat intestine acetone powder was purchased and used to prepare enzyme solution according to the method of Oki et al. [53] with slight modifications. In brief, rat intestinal acetone powder (25 mg/ml) was homogenized in ice-cold 50 mM phosphate buffer. After centrifugation at 6 000 × g for 15 min, the clear supernatant obtained was used as the enzyme solution for determining α-glucosidase activity.

The rat intestinal sucrase inhibitory activity was determined using the method of Bhandari et al. [54], with a slight modification. Sucrose (200 μl of a 56 mM solution) in 0.1 M potassium phosphate buffer (pH 7) was mixed with 100 μl of crude acetone extracts (2.5 mg/ml) in 50% aqueous dimethyl sulphoxide (DMSO) in a test tube. After pre-incubation at 37°C for 5 min, 200 μl of rat intestinal α-glucosidase solution prepared as described above was added. Whereas 100 μl DMSO (50%) was used in place of the plant extract to represent 100% enzyme activity, Acarbose (0.1 mg/ml) was used as the positive control and for the blanks (negative controls) the enzyme solution was replaced with distilled water and the same procedure was carried out as above. Final concentrations in the incubation mixture for plant extracts were 0.5 mg/ml and 0.02 mg/ml for acarbose.

After thoroughly mixing, the test sample, solvent and positive control test tubes were incubated at 37°C for 20 min and then the reaction was stopped by adding 750 μl of 2 M Tris–HCl buffer (pH 6.9). The amount of liberated glucose was determined by the glucose oxidase method using a commercial test kit (Sigma GAGO 20) according to the manufacturer’s instructions. The absorbance of the wells was measured at 540 nm and the inhibitory activity was calculated using the following formula:

$$\begin{array}{l} \%\;\mathrm{Inhibition}=100\times \left(\frac{\Delta A_{\mathrm{Control}}-\Delta A_{\mathrm{Sample}}}{\Delta A_{\mathrm{Control}}}\right)\\ \;\Delta A_{\mathrm{Control}}=A_{\mathrm{Test}}-A_{\mathrm{Blank}}\\ \;\Delta A_{\mathrm{Sample}}=A_{\mathrm{Test}}-A_{\mathrm{Blank}} \end{array}$$

### Kinetics of inhibition against α- amylase and α- glucosidase activities

The kinetics of inhibition of the crude acetone extract of *F. lutea* against α- amylase and α- glucosidase activities were measured by increasing substrate concentrations of starch (0.016% - 0.5%) and sucrose (7 mM – 56 mM) respectively in the absence and presence of extract of *F. lutea* at concentrations of 25–100 μg/ml for α-amylase inhibitory assay and 62.5 – 250 μg/ml for α- glucosidase inhibitory assay. The type of inhibition was determined by Lineweaver-Burk double reciprocal plot analysis of the data, which was calculated from the result according to Michaelis-Menten kinetics [11].

### Calculation of EC50

A dose response effect was conducted on α- amylase and α-glucosidase for the extracts. The concentrations of extracts used ranged from 16 to 500 μg/ml. The concentration of extract that inhibited 50% of the enzyme activity under assay conditions was defined as the effective concentration (EC50) and this was established using the programme Kinetica 5 (Thermo). The concentration response relationship was plotted and fitted to a Hill’s model. The model was described by the following equation; $y=\frac{y_{\max}x^{\propto }}{c^{\propto }+x^{\propto }}$. The EC50 concentrations attained were used for comparison of the potency of the various crude acetone plant extracts.

### Statistical analysis

All experiments were performed in triplicate and repeated three times to yield nine dose- response curves. Data are presented as the mean ± standard error of mean (S.E.M.). Statistical significances were evaluated by one-way analysis of variance (ANOVA) and student’s *t*-test, with *post hoc* analysis using the Bonferroni corrected *t*-test. *P*-values less than 0.05 were considered to be significant.

## Results

### Extraction of plant material

Ten *Ficus* species were selected on the basis of availability and were extracted with acetone. In Table 3, the percentage yields of crude acetone extracts of the ten *Ficus* species are reported. The percentage yield ranged from 2.32% to 3.70% among the leaf extracts where the extract of *F. lutea* had the highest yield (3.70%), followed by *F. polita* (3.15%). The extracts of *F. natalensis* (2.35%) and *F. capreifolia* had the lowest yields (2.32%).

**Table 3:** **Percentage yield, total polyphenol content and antioxidant activity of crude acetone extracts of leaves of ten *Ficus *species**

**Species**	**Yield (%)**	^**a**^**Total polyphenol** **(mg GAE/g dry weight)**	^**ab**^**Antioxidant activity** **TEAC**
*Ficus capreifolia*	2.32	4.73 ± 0.26^c^	0.34 ± 0.05^c^
*Ficus cordata*	2.65	8.23 ± 1.00^d^	0.27 ± 0.03^c^
*Ficus craterostoma*	2.60	9.80 ± 0.93^d^	0.66 ± 0.06^d^
*Ficus glumosa*	2.60	19.24 ± 0.79^e^	1.29 ± 0.30^e^
*Ficus lutea*	3.70	56.85 ± 1.82^f^	4.80 ± 0.90^f^
*Ficus natalensis*	2.35	4.75 ± 0.92^c^	0.69 ± 0.08^d^
*Ficus polita*	3.15	8.04 ± 0.52^d^	0.31 ± 0.06^c^
*Ficus religiosa*	2.45	5.40 ± 0.35^c^	0.59 ± 0.18^c^
*Ficus sycomorus*	2.60	12.33 ± 0.26^e^	1.91 ± 0.19^e^
*Ficus thonningii*	2.40	4.64 ± 0.48^c^	0.77 ± 0.06^d^

^a^Values are means of triplicate determinations done three times (n = 9) ± standard error;

^b^Antioxidant activity (Trolox equivalent antioxidant capacity).

^c,d,e,f^No significant difference between extracts with same value, but significant difference *p*<0.05 between different values.

### Total polyphenol content and antioxidant activity

The total polyphenol content of the crude acetone extracts of the ten *Ficus* species has highly variable (Table 3), with a range of 4.64 to 56.85 mg GAE/g dry weight of plant. When the total polyphenol content of each extract was compared, the extract of *F. lutea* (56.85 ± 1.82 mg/g) was found to have a remarkably higher content (*p*˂0.001) followed in decreasing order by extracts of *F. glumosa* and *F. sycomorus* with total polyphenol content of 19.24 ± 0.79 and 12.33 ± 0.26 mg GEA/g dry weight of plant respectively. The extracts with the lowest values in decreasing order were *F. natalensis, F. capreifolia* and *F. thonningii* with total phenolic content of 4.75 ± 0.92, 4.73 ± 0.26 and 4.64 ± 0.28 mg GAE/g dry weight of plant respectively.

The antioxidant activities of the extracts, expressed as Trolox equivalent antioxidant capacity (TEAC) are listed in Table 3. The crude acetone extracts of the ten *Ficus* species had different antioxidant capacities (Table 3). The total antioxidant activity for the ten *Ficus* species varied widely from 4.80 ± 0.90 to 0.27 ± 0.03 TEAC under the assay conditions. The antioxidant activity of the extract of *F. lutea* (4.80 ± 0.90 TEAC) was significantly different (*p*˂0.001) when compared with that of the other extracts, followed by *F. sycomorus* and *F. glumosa* in decreasing order with antioxidant activity of 1.91 ± 0.18 and 1.29 ± 0.30 TEAC respectively. The extracts with the lowest TEAC in decreasing order were *F. capreifolia*, *F. polita* and *F. cordata* with 0.34 ± 0.05, 0.31 ± 0.06 and 0.27 ± 0.03 TEAC respectively. The correlation coefficient between total polyphenolic content and TEAC of the crude acetone extracts of the ten *Ficus* species R^2^ was 0.50.

### α-Amylase inhibitory activity of extracts of *Ficus* species

The inhibitory activity of the crude acetone extracts of the ten *Ficus* species are presented in Table 4. The extract of *F. lutea* had the highest inhibitory potential with activity of 95.4 ± 1.2% inhibition at 1 mg/ml. This was followed by the extract of *F. glumosa* with 65.1 ± 3.0% inhibition at the same concentration while Acarbose, the positive control, had inhibitory activity of 96.7 ± 0.3% at 0.04 mg/ml. The extracts of the other *Ficus* species moderately inhibited α-amylase with activity between 40% and 45%, with the exception of the extracts of *F. religiosa* and *F. thonningii* which had poor inhibitory activities. The EC50s are presented in Table 4. The extract of *F. lutea* was most potent (EC50 of 9.42 ± 2.01 μg/ml) followed by *F. craterostoma* (EC50 = 11.41 ± 4.68 μg/ml) and *F. natalensis* (EC50 = 17.85 ± 4.42 μg/ml) with no significant difference present between them (*p*<0.05*).* Acarbose was characterised by an EC50 of 0.04 ± 0.03 μg/ml. The correlation coefficient between total polyphenolic content and inhibition of α-amylase activity by the extracts of the ten *Ficus* species R^2^ was 0.65. Conversely, the correlation coefficient between antioxidant activity (TEAC) and inhibition of α-amylase activity by the crude acetone extracts of the ten *Ficus* species R^2^ was 0.41.

**Table 4:** **The percentage inhibition of α-amylase activity (1 mg/ml) and concentration leading to 50% inhibition (EC50) of crude acetone extracts of the ten selected *Ficus *species**

**Species**	**(%) α- amylase inhibition**^**a**^	**EC50 (μg/ml)**
*Ficus capreifolia*	43.8 ± 3.3^b^	>100
*Ficus cordata*	45.9 ± 5.2^b^	> 100
*Ficus craterostoma*	48.3 ± 1.6^b^	11.41 ± 4.68^b^
*Ficus glumosa*	65.1 ± 3.0^c^	> 100
*Ficus lutea*	95.4 ± 1.2^d^	9.42 ± 2.01^b^
*Ficus natalensis*	43.7 ± 1.8^b^	17.85 ± 4.42^b^
*Ficus polita*	40.2 ± 2.6^b^	> 100
*Ficus religiosa*	35.3 ± 2.8^b^	> 100
*Ficus sycomorus*	40.0 ± 2.8^b^	> 100
*Ficus thonningii*	37.6 ± 2.7^b^	> 100
Acarbose	96.7 ± 0.3	0.04 ± 0.03

^a^% α-amylase inhibitory activity of crude acetone extracts of ten South African *Ficus* species (1 mg/ml) and control (acarbose) (0.04 mg/ml). Results expressed as% mean ± SEM (n = 9). ^b,c,d^No significant difference between extracts with same value, but significant difference *p*<0.05 between different values.

### α-Glucosidase inhibitory activity of extracts of *Ficus* species

The crude acetone extracts of the ten *Ficus* species were generally weak inhibitors of α- glucosidase activity (Table 5), with only *F. lutea* demonstrating inhibitory activity of 64.3 ± 3.6% at a concentration of 0.5 mg/ml (Significantly different (*p*˂0.05) to the other extracts). Acarbose (positive control) was a potent inhibitor of α-glucosidase (84.8 ± 1.7%) at a concentration of 0.02 mg/ml. The EC50s for the inhibitory activity is presented in Table 5. The extract of *F. sycomorus* was most potent with an EC50 of 217 ± 69 μg/ml, followed closely by the extract of *F. lutea* (290 ± 111 μg/ml), with no significant difference between them (*p*˂0.05). The EC50 of Acarbose was 3.4 ± 0.5 μg/ml. The correlation coefficient between total polyphenolic content and inhibition of α-glucosidase activity by the crude acetone extracts of the ten *Ficus* species R^2^ was 0.74. In addition, the correlation coefficient between antioxidant activity (TEAC) and inhibition of α-glucosidase activity by the crude acetone extracts of the ten *Ficus* species R^2^ was 0.67.

**Table 5:** **The percentage inhibition of α-glucosidase activity (0.5 mg/ml) and concentration leading to 50% inhibition (EC50) of crude acetone extracts of ten *Ficus *species**

**Species**	**(%) α-glucosidase inhibition**^**a**^	**EC50 (μg/ml)**
*Ficus capreifolia*	24.3 ± 1.7^b^	> 1000
*Ficus cordata*	22.0 ± 3.6^b^	> 1000
*Ficus craterostoma*	28.2 ± 7.0^b^	> 1000
*Ficus glumosa*	38.7 ± 6.5^b^	> 1000
*Ficus lutea*	64.3 ± 3.6^c^	290 ± 111^b^
*Ficus natalensis*	23.6 ± 8.1^b^	> 1000
*Ficus polita*	29.2 ± 5.8^b^	> 1000
*Ficus religiosa*	17.6 ± 8.0^b^	> 1000
*Ficus sycomorus*	35.7 ± 5.4^b^	217 ± 69^b^
*Ficus thonningii*	25.3 ± 5.0^b^	> 1000
Acarbose	84.8 ± 1.7	3.4 ± 0.5

^a^% α-glucosidase inhibitory activity of crude acetone extracts of ten South African *Ficus* species (0.5 mg/ml) and control (acarbose) (0.02 mg/ml). Results expressed as % mean ± SEM (n = 9).

^b,c.^No significant difference between extracts with same value, but significant difference *p*<0.05 between different values.

### The enzyme kinetics of α- amylase and α- glucosidase inhibition by extract of *F. lutea*

**Figure 1:**
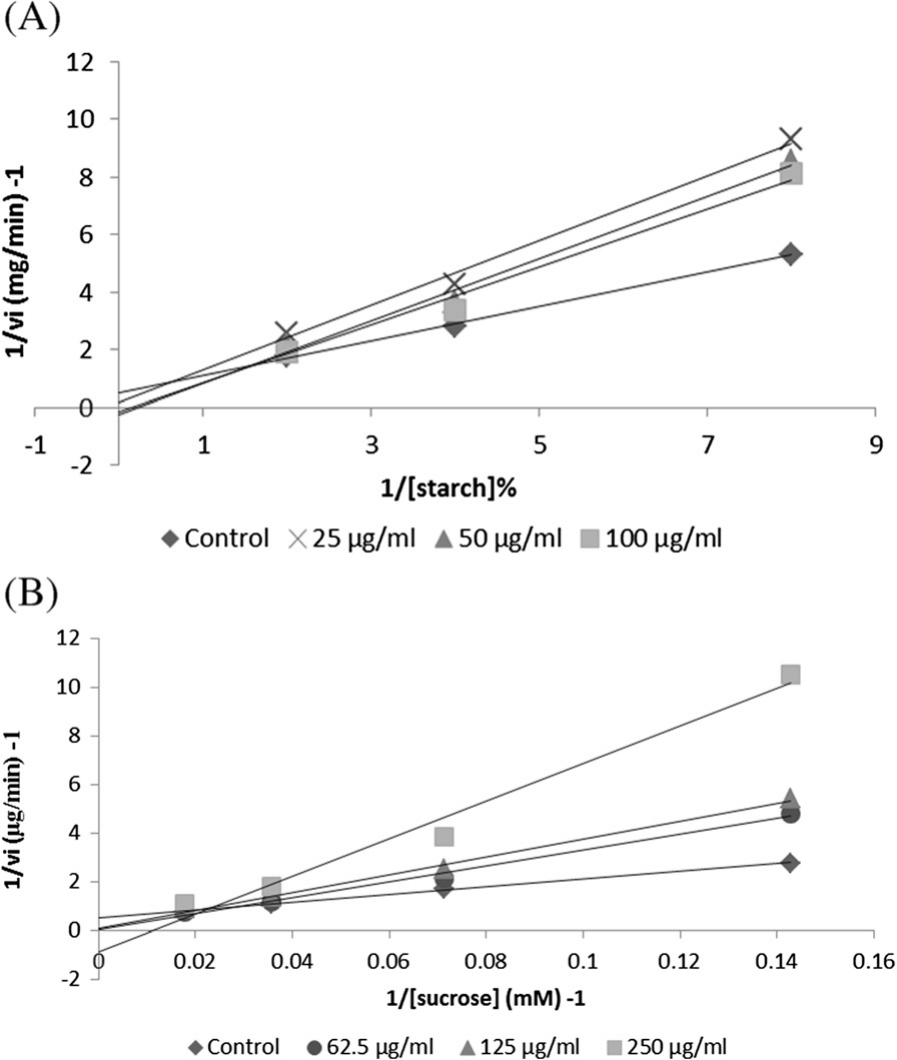
**Lineweaver-Burk double reciprocal plots for kinetic analysis of the reaction of porcine pancreatic α-amylase with starch (%) used as substrate (A) and rat intestinal α-glucosidase with sucrose (mM) used as substrate (B) in the absence and presence of extract of *F. lutea.***

Analysis of the α-amylase and α-glucosidase kinetics by the crude acetone extract of *F. lutea* is shown in Figure 1. For the α-amylase (Figure 1A) and α-glucosidase (Figure 1B) inhibition by *F. lutea*, the intersection of the double reciprocal plot is seated at a point above the +1/[s] axis, indicating that *F. lutea* acts as a partially non-competitive-type inhibitor of α-amylase and α- glucosidase.

## Discussion

The most challenging goal in the management of type 2 diabetes mellitus is to achieve blood glucose levels as close to normal as physiologically possible [55]. With postprandial hyperglycaemia being the earliest metabolic abnormality detectable, modulation of this rise is an important tool in the management of the diabetic patient [56,57]. α-Amylases are endoglucanases, which hydrolyse the internal α-1,4 glucosidic linkages in starch, and α- glucosidase (sucrase), one of the glucosidases located in the brush border surface membrane of intestinal cells, is a key enzyme for carbohydrate digestion and absorption. These enzymes have been recognized as therapeutic targets for modulation of postprandial hyperglycaemia. In addition, prolonged hyperglycaemia is an independent risk factor for the development of microvascular and macrovascular complications of diabetes mellitus which is mediated through oxidative stress [55,58].

In this study the inhibitory effectiveness of the acetone extract of the leaves of ten *Ficus* species against the α-amylase and α-glucosidase enzymes were evaluated. As a secondary objective, the relationship between total polyphenolic content and antioxidant activity was also studied. For this study, a positive correlation between total polyphenolic content and inhibition of α-amylase and α-glucosidase activity was present with R^2^ values of 0.65 and 0.74 respectively. This therefore indicates the likelihood of the polyphenols in the acetone extracts being partly responsible for the inhibition of the activity of the enzymes. The latter was not an unexpected finding as the polyphenolic extracts from a number of plants have been previously shown to be effective inhibitors of the intestinal α-glucosidase and α-amylase enzyme systems [59].

The extracts of ten *Ficus* species were evaluated for their potential to specifically inhibit activity of porcine pancreatic α-amylase and rat small intestinal α-glucosidase. The ten *Ficus* species were generally potent inhibitors of activity of the porcine pancreatic α-amylase but weak inhibitors of activity of the rat small intestinal α-glucosidase. Three of the four *Ficus* species with *in vivo* blood glucose lowering potential in previous studies (Table 2) were all weak inhibitors of the activity of α-amylase and α-glucosidase *in vitro*. While the reason for the difference could be due to the different compounds being extracted as a result of different solvents used in these studies, the more likely reason was that the glucose lowering potential of these plants may be due to non-α-amylase or α-glucosidase inhibitory activities, such as the stimulation of glucose uptake, the stimulation of insulin release or the inhibition of gluconeogenesis.

The fourth *Ficus* species (*F. sycomorus*) which is used traditionally by the Hausa and Fulani tribes of northern Nigeria to treat diabetes [33] together with known glucose lowering potential *in vivo* (Table 2) was a weak inhibitor of α-amylase activity but a potent inhibitor of α-glucosidase (sucrase) activity with an EC50 of 217 ± 69 μg/ml. We therefore believe that the glucose lowering potential demonstrated *in vivo* by the extract of *F. sycomorus* may be due to the inhibition of α-glucosidase activity (sucrase). Some authors have shown that the extracts of other *Ficus* species such as *F. racemose*[60], *F. benghalensis*[61] and *F. deltoidea*[62] inhibit the activity of sucrase with an EC50 of 367 ± 15.2 μg/ml and 239 ± 14.3 μg/ml respectively for cold and hot water extract of *F. racemosa*, and 193 ± 21.6 μg/ml and 141 ± 22.1 μg/ml respectively for cold and hot water extract of *F. benghalensis*. This was in agreement with our results. Furthermore, studies also showed the activity of α-amylase is inhibited with an EC50 of 0.94 ± 0.15% and 0.58 ± 0.15% respectively for the cold and hot water extracts of *F. racemosa,*[60] and, 4.4 and 125 μg/ml respectively for the cold and hot water extracts of *F. benghalensis*[63], this was also in agreement with our results.

Some studies have observed the potency with which polyphenols inhibited the activity of porcine pancreatic α-amylase and rat small intestinal α-glucosidase to be different [59,64]. Strawberry and raspberry extracts were the most effective inhibitors of α-amylase followed by blueberry and blackcurrant. Although these extracts also inhibited rat intestinal α- glucosidase activity, the order of effectiveness was different than with α-amylase. Blueberry and blackcurrant were the most effective followed by strawberry and raspberry [59]. Rat intestinal α-glucosidase is generally weakly inhibited by many flavonoids, while flavonoids are often potent inhibitors of porcine pancreatic α-amylase [64]. It is possible that the extracts of the *Ficus* species used in this study demonstrated potent α-amylase inhibitory activity because they contain more of the flavonoid groups with the extract of *F. lutea* being the most potent.

Polyphenolic compounds may also be indirectly beneficial in disease by chelating metal ions [65] or activating the expression of antioxidant enzymes [66] or directly acting as antioxidants [67]. High total phenolic contents may not always translate to a high antioxidant activity. Pulido et al. [68] reported that antioxidant efficiency of the polyphenols seemed to depend on the position and extent of hydroxylation and conjugation. This was also observed in this study where the correlation between total polyphenolic content and antioxidant activity had an R^2^ value of 0.5. We found that extracts of *F. glumosa, F. sycomorus* and *F. lutea* had good antioxidant activities and high total phenolic contents. Some authors have shown that *F. glumosa*[30], *F. sycomorus*[69] and *F. lutea*[39] have very good DPPH^**.**^ scavenging activities with reported IC50 of 79.5 ± 1.77 μg/ml and 11.9 ± 0.3 μg/ml respectively for *F. sycomorus* and *F. lutea*, and this is in agreement with our results. Among these three *Ficus* species, the extract of *F. lutea* displayed the highest total polyphenolic content and the highest antioxidant activity.

In addition, the qualitative phytochemical analysis conducted (results not included) showed that tannin was one of the prominent phytochemicals contained in the crude acetone extract of *F. lutea*. Hagerman et al. [70] reported that the high molecular weight polyphenols (tannins) have the ability to quench free radicals (ABTS^+^) and their effectiveness depends on the molecular weight, the number of aromatic rings and the nature of the hydroxyl group substitutions. It is also possible that not all polyphenolic compounds possess ABTS ^+^ radical scavenging activities [71]. Free radical (ABTS^+^) scavenging of *Ficus* species may be due to the presence of high molecular weight polyphenols, such as catechins, pelargonidins and leucopelargonidin derivatives in addition to the flavonoids [72]. A study of the correlation between antioxidant activity and inhibition of α-amylase and α-glucosidase activity by the ten *Ficus* species had an R^2^ value of 0.41 and 0.67 respectively indicating that the polyphenolic compounds responsible for antioxidant activity may likely be responsible for the inhibition of α-glucosidase activity but may not be responsible for the inhibition of α-amylase activity. Some authors have demonstrated that compounds with potent antioxidant activity are also strong inhibitors of α-glucosidase activity.

When studying the kinetics of inhibition, the extract of *F. lutea* showed partial non- competitive inhibition against porcine pancreatic α-amylase and α-glucosidase. A fully non- competitive inhibitor binds the enzyme substrate [ES] complex, not the free enzyme and affects the breakdown of the [ES] to form a product. Likewise, a partial non-competitive inhibitor binds to the enzyme-substrate complex but it is assumed that the inhibitor is released from the enzyme at the same time [ES] is broken down to the product [73]. This type of inhibition does occur where there are multiple inhibitors as will be the case for a crude extract of *F. lutea*[73]. Another study demonstrated that pure compounds such as D-xylose and (+)- catechin are un-competitive inhibitors of rat intestinal α-glucosidase activity [53].

The results of this study revealed that the crude acetone extract of *F. lutea* is high in total polyphenolic content and antioxidant activity, and is a potent inhibitor of α-amylase activity. This *in vitro* study has demonstrated that polyphenolic compounds present in *F. lutea* may likely be responsible for the inhibitory activity against the carbohydrate hydrolysing enzymes.

## Conclusion

This study investigated the potential antidiabetic activity of ten South African *Ficus* species, focussing on effects on digestive enzymes, polyphenolic content and antioxidant activity. All acetone extracts of the ten *Ficus* species had α-amylase and α-glucosidase enzyme inhibitory activity. The crude acetone extract of *F. lutea* was the most active in all the assays. This is the first report of the antidiabetic potential of *F. lutea* in potentially lowering blood glucose levels *in vitro*. The crude acetone extract of *F. lutea* was more active *in vitro* than the crude acetone extracts of the three *Ficus* species that are reported to be used traditionally to treat diabetes. The activity that is responsible for the antidiabetic activity appears to reside in the polar fraction of the extract of *F. lutea*, so rural people should be able to extract the active compound(s) with water or alcohol. Research is underway to isolate the active compound responsible for the antidiabetic activity, to confirm the *in vitro* antidiabetic activity, and to investigate toxicity and efficacy *in vivo*.

## Competing interests

The authors declare that they have no competing interests.

## Authors’ contributions

OOO carried out the study and wrote the manuscript; VN and JNE contributed to conception, design, analysis and interpretation of data, and LJM, JNE and VN assisted with and supervised the manuscript writing. All authors have read and approved the final manuscript.

## Pre-publication history

The pre-publication history for this paper can be accessed here:

http://www.biomedcentral.com/1472-6882/13/94/prepub
